# Supplementary material for: 3, 3′- (3, 5-DCPBC) Down-Regulates Multiple Phosphokinase Dependent Signal Transduction Pathways in Malignant Melanoma Cells through Specific Diminution of EGFRY1086 Phosphorylation
Source: Molecules. 2022 Feb 9;27(4):1172. doi: 10.3390/molecules27041172 (PMC8874408; doi:10.3390/molecules27041172)
Supplement: Supplementary file 1 [file molecules-27-01172-s001.zip › molecules-1496558-supplementary.pdf]

Table S1. Non affected kinases of proteome profiling assay at 4 hrs.

Protein Kinases	Phosphorylation Site	Statistical Analysis<br>* ϱ ≤0.05<br>** ϱ≤0.005<br>***ϱ≤0.0005
**p38alpha**	T180/Y182	0.33
**JUNK1/2/3**	T183/Y187/T185/Y185<br>/221/223	0.06
**AMPKα1**	T183	0.14
**AKT1/2/3**	S473	0.09
**AMPKα2**	T172	0.08
**STAT5β**	Y699	0.06
**AKT1/2/3**	T308	0.66
**P70S6KINASE**	T389	0.35
**P53**	S15	0.09
**C JUN**	S63	0.29
**P70S6KINASE**	T421/S424	0.09
**RSK1/2/3**	S380/S386/S377	0.96
**ENOS**	S1177	0.53
**STAT3**	Y705	0.27
**P27**	T198	0.66
**PLCγ1**	Y783	0.66
**STAT3**	S727	0.33
**WNK1**	T60	0.72
**PYK2**	Y402	0.86
**SHP60**		0.89

Abbreviations; Threonine: T, Tyrosine: Y, Serine: S.

Supplementary Table S1. Table shows the list of genes (left column) and various phosphorylation sites that were associated with transcription factors (middle column) and the ratio of the phosphorylation levels of A375 MM cells treated with 3,5-DCPBC *vs* DMSO in arbitrary units for a period of 4 hrs (right column).

Table S2. Non affected kinases of proteome profiling assay at 18 hrs.

Protein Kinases	Phosphorylation Sites	Statistical Analysis<br>* ϱ ≤0.05<br>** ϱ≤0.005<br>***ϱ≤0.0005
**p38alpha**	T180/Y182	0.58
**ERK1/2**	T202/T185/Y187/Y204	0.70
**EGFR**	Y1086	0.12
**AKT1/2/3**	S473	0.90
**mTOR**	S2448	0.60
**SHP27**	-	0.96
**AMPK**α**2**	T172	0.60
**β -Catenin**	-	0.54
**Src**	Y419	0.43
**Lyn**	Y397	0.21
**Lyk**	Y394	0.15
**STAT2**	Y689	0.74
**STAT5**α	Y697	0.57
**Fyn**	Y420	0.41
**Fgr**	Y412	0.84
**STAT6**	Y641	0.51
**Hck**	Y411	0.52
**STAT5**α**/β**	Y694,Y699	0.13
**FAK**	Y397	0.87
**Chk-2**	T68	0.80
**PDGFB**	Y751	0.44
**STAT5**α**/β**	Y694,Y699	0.97
**PRAS40**	T246	0.13
**P53**	S392	0.86
**P53**	S15	0.91
**P53**	S46	0.46
**AKT1/2/3**	T308	0.76
**P70S6KINASE**	T389	0.32
**C JUN**	S63	0.34
**P70S6KINASE**	T421/S424	0.16
**RSK1/2/3**	S380/S386/S377	0.80
**ENOS**	S1177	0.23
**STAT3**	Y705	0.40
**P27**	T198	0.16
**PLC**γ**1**	Y783	0.36
**STAT3**	S727	0.55
**WNK1**	T60	0.45

**PYK2**	Y402	0.41
**SHP60**	-	0.40

Supplementary Table S2. Table shows the list of the genes (left column) and various phosphorylation sites that were associated with transcription factors (middle column) and the ratio of the phosphorylation levels of A375 MM cells treated with 3,5-DCPBC vs DMSO in arbitrary units for a period of 18 hrs (right column).
